# Comparative Genomic Analysis of the Hydrocarbon-Oxidizing Dibenzothiophene-Desulfurizing *Gordonia* Strains

**DOI:** 10.3390/microorganisms11010004

**Published:** 2022-12-20

**Authors:** Ekaterina Frantsuzova, Yanina Delegan, Alexander Bogun, Diyana Sokolova, Tamara Nazina

**Affiliations:** 1 Institute of Biochemistry and Physiology of Microorganisms, Federal Research Center “Pushchino Scientific Center for Biological Research of Russian Academy of Sciences” (FRC PSCBR RAS), 142290 Pushchino, Russia; 2 State Research Center for Applied Microbiology and Biotechnology, 142279 Obolensk, Russia; 3 Winogradsky Institute of Microbiology, Research Center of Biotechnology, Russian Academy of Sciences, 119071 Moscow, Russia

**Keywords:** *Gordonia*, genome assembly, hydrocarbon-oxidizing bacteria, dibenzothiophene, biodegradation

## Abstract

A number of actinobacteria of the genus *Gordonia* are able to use dibenzothiophene (DBT) and its derivatives as the only source of sulfur, which makes them promising agents for the process of oil biodesulfurization. Actinobacteria assimilate sulfur from condensed thiophenes without breaking the carbon–carbon bonds, using the 4S pathway encoded by the dszABC operon-like structure. The genome of the new dibenzothiophene-degrading hydrocarbon-oxidizing bacterial strain *Gordonia amicalis* 6-1 was completely sequenced and the genes potentially involved in the pathways of DBT desulfurization, oxidation of alkanes and aromatic compounds, as well as in the osmoprotectant metabolism in strain 6-1 and other members of the genus *Gordonia*, were analyzed. The genome of *G*. *amicalis* strain 6-1 consists of a 5,105,798-bp circular chromosome (67.3% GC content) and an 86,621-bp circular plasmid, pCP86 (65.4% GC content). This paper presents a comparative bioinformatic analysis of complete genomes of strain 6-1 and dibenzothiophene-degrading *Gordonia* strains 1D and 135 that do not have the dsz operon. The assumption is made about the participation in this process of the region containing the *sfn*B gene. Genomic analysis supported the results of phenomenological studies of *Gordonia* strains and the possibility of their application in the bioremediation of oil-contaminated environments and in the purification of oil equipment from oil and asphalt-resin-paraffin deposits.

## 1. Introduction

Condensed thiophenes may be responsible for most of the sulfur content in crude oil. Their presence results in lower oil quality and increased cost of processing; they also promote occurrence of acid rains and air contamination due to sulfur dioxide release from burning fuel [1]. Hydrodesulfurization processes, which are often used in oil refineries for sulfur removal, are economically inefficient. Biological desulfurization proceeds at low temperature and pressure and represents an inexpensive method alternative to hydrodesulfurization [2]. Biodesulfurization of organosulfur compounds usually involves the microorganisms utilizing sulfur via the 4S pathway, which is known to be active in a number of genera, including *Stenotrophomonas* [3], *Rhodococcus* [4,5,6], *Gordonia* [6,7,8], *Bacillus* [9], *Shewanella* [10], and others.

Dibenzothiophene (DBT), a heterocyclic organosulfur compound consisting of three condensed cycles, is used as a model compound for investigation of biodesulfurization of organic compounds. Application of *Gordonia* and other bacterial genera for biodesulfurization makes it possible to decrease the content of the oil organosulfur compounds, including DBT and its alkylated derivatives, which are not affected by physicochemical desulfurization [11]. Sulfur removal from polycyclic aromatic sulfur-containing hydrocarbons is carried out under mild conditions, involves low operating costs, and results in decreased release of greenhouse gases compared to hydrodesulfurization.

The genus *Gordonia* comprises aerobic, Gram-positive, non-spore-forming actinobacteria of the family *Gordoniaceae*, order Corynebacteriales [12]. At the time of composing the manuscript (November 2022), the genus contained over 50 validly described species [13] isolated from various environments. Members of the genus *Gordonia* are known for their remarkable catabolic activities and may use a broad range of aliphatic and aromatic hydrocarbons, polycyclic aromatic hydrocarbons (PAHs), alkylpyridines, phthalates, xenobiotics, crude oil and diesel fuel [14,15,16,17,18,19,20,21]. For example, *Gordonia* sp. strain IITR100 isolated from petroleum-contaminated soil could utilize 4,6-dimethyl dibenzothiophene as the sole sulfur source [22,23] and desulfurize 76% sulfur from heavy crude oil, which resulted in a 31% decrease of the oil viscosity [24]. The strain was also able to produce biosurfactants [25]. Thus, *Gordonia* strains could be applied for enhancement of oil recovery from oil-bearing rocks.

The ability to grow on thiophenes has been found in many *Gordonia* strains [26,27]. This feature has been observed in members of the species *G. amicalis* [14], *G. alkanivorans* [28], *G. terrae* [29], *G. aichiensis* [30], *G. desulfuricans* [31].

There are also several examples of DBT-degrading *Gordonia* that have not been taxonomically identified [15,32,33]. The growth on thiophenes in *Gordonia* strains does not always mean that these compounds are used as the sole source of sulfur. For example, *Gordonia* sp. strain 213E (NCIMB 40816) desulfurizes benzothiophene but is unable to grow in a mineral medium with DBT as the sole sulfur source [7]. In contrast, the strains *Gordonia* sp. F.5.25.8 [33], *G*. *alkanivorans* RIPI90A [27], and *G*. *amicalis* DSM 44461^T^ [14] utilize DBT as the sole sulfur source in the culture medium.

Gupta and co-workers [34] distinguish the following major pathways for bacterial degradation of DBT: (1) 4S-pathway [35], (2) carbon–carbon destruction pathway, which produces sulfur and (3) Kodama pathway [36]. Until recently, it was believed that the process of DBT degradation in actinobacteria occurs only under the control of the dsz operon [26,28,30]. Genes of the DBT desulfurization gene cluster (dsz operon) were revealed in genomes of eight *Gordonia* strains by the BLAST service.

We have previously isolated two *Gordonia* strains, *Gordonia amicalis* 1D and *Gordonia alkanivorans* 135, which utilize DBT as the sole sulfur source for growth [37,38,39]. Genome sequencing showed that these strains had no dsz operon, required for the 4S pathway of thiophene desulfurization. A new DBT-desulfurizing hydrocarbon-oxidizing *Gordonia amicalis* strain 6-1 was isolated from Cheremukhovskoe heavy oil reservoir (Republic of Tatarstan, Nurlat, Russia) [40,41]. The aim of this study was the whole genome sequencing of *G amicalis* 6-1 and comparison of the gene clusters responsible for dibenzothiophene desulfurization and alkane degradation in genomes of strain 6-1 and of two previously isolated strains *G. amicalis* 1D and *G. alkanivorans* 135 that had no dsz gene cluster. We show that DBT degradation is also possible under the control of other genes and suggest that the region containing the *sfn*B gene may determine DBT desulfurization. Genomic analysis confirmed phenomenological observations of the ability of *Gordonia amicalis* 6-1 to grow on *n*-alkanes and aromatic substrates and to participate in biodesulfurization of organosulfur compounds of oil and the prospect of biotechnological application of strains of the genus *Gordonia* in environmental technologies.

## 2. Materials and Methods

### 2.1. Phenotypic Characterization of Gordonia amicalis Strain 6-1

Hydrocarbon-oxidizing bacterium *G. amicalis* strain 6-1 was isolated from production water reinjected into Cheremukhovskoe oilfield, Republic of Tatarstan, Russia. Physiological characteristics of strain 6-1 and the spectrum of utilized substrates were described previously [40]. The cells of strain 6-1 are Gram-positive non-spore-forming, nonmotile rods (Figure S1). On the Plate Count Agar (PCA, Merck, Darmstadt, Germany) medium the strain forms small round mucous colonies of orange color. The strain grows at a temperature range from 5 to 45 °C (optimum 28 °C); in the pH range from 6.5 to 8.2 (optimum 7.4), and in the presence of 0–10% (*w*/*v*) NaCl (optimum 1.5–7%). Based on the high 16S rRNA gene sequence similarity (99.3%) of strain 6-1 (acc. number MN101280) with the gene of the type strain *Gordonia amicalis* IEGM^T^ [14], the strain 6-1 was assigned to this species. Strain *G*. *amicalis* 6-1 aerobically utilizes sugars (sucrose, glucose, arabinose), amino acids (alanine, glutamic acid), alcohols (methanol, ethanol, glycerol), and acetic, lactic, and propionic acids as the only carbon and energy sources, as well as C_11_, C_12_, C_13_, C_15_–C_21_, and C_23_ *n*-alkanes of crude oil and diesel fuel. Oil biodegradation by strain 6-1 is accompanied by biosurfactants production, decreasing the surface tension of the bacteria-free culture liquid to 23.7 mN/m and by changes in the oil composition. The content of mono- and bicyclic aromatic hydrocarbons decreased, while the content of resins increased [40]. The strain 6-1 grew in a medium with glucose, using organosulfur aromatic compounds (dibenzothiophene) as a source of sulfur [41].

### 2.2. DNA Isolation, Genome Sequencing and Assembly

Aiming at a better understanding of the genetic information determining the hydrocarbon oxidation and DBT desulfurization by strain *G. amicalis* 6-1, its whole genome was sequenced. Strain 6-1 was grown on LB agar at 28 °C. Cells were harvested, and genomic DNA was isolated using a DNeasy Blood & Tissue Kit (catalog number 69506, QIAGEN, Germantown, MD, USA) The full genome sequence of strain 6-1 was determined using Illumina technology (Illumina Inc, San Diego, CA, USA) and monomolecular nanopore sequencing (Oxford Nanopore, Didcot, UK). Sequencing of strain 6-1 was carried out at the Institute of Biochemistry and Physiology of Microorganisms, Federal Research Center “Pushchino Scientific Center for Biological Research of Russian Academy of Sciences” using a MinION nanopore sequencing with the R9.4.1 flow cell (Oxford Nanopore Technologies, Didcot, UK). The library was prepared using the Ligation Sequencing kit 1D (SQK-LSK109) according to the manufacturer’s protocol (Oxford Nanopore Technologies, Didcot, UK). Genomic DNA was also sequenced on the Illumina NovaSeq 6000 platform (BioSpark, Troitsk, Russia) using the S2 reagent kit and Kapa HyperPlus kit (Roche, Wilmington, NC, USA) for the preparation of libraries. The Illumina and MinION Nanopore reads were used for hybrid assembly with the SPAdes software package (version 3.13.1, St. Petersburg, Russia) [42]. The MinION nanopore sequencing reads were assembled into contigs using the Flye software package (2.9.1 release, San Diego, CA, USA) [43] and combined into replicons for further use as the standard sequences. The Illumina reads were used to correct the errors of nanopore sequencing and assembly using the Bowtie2 software package (version 2.5.0, Baltimore, MD, USA) [44].

### 2.3. Genome Analysis

Annular maps of the chromosomes and plasmids of *G. amicalis* strain 6-1 and of strains D1 and 135 used for comparison were constructed using the Proksee web service [https://proksee.ca/, accessed on 10 October 2022]. Average nucleotide identity (ANI) of the obtained plasmid was compared to the plasmids of closely related type strains [45]. Structural annotation of the genomes and search for the target genes presumably involved in DBT degradation were carried out using the Prokka (https://doi.org/10.1093/bioinformatics/btu153, v.1.14.5, accessed on 29 November 2019) [46], RAST (https://rast.nmpdr.org/rast.cgi, v. 2.0, accessed on 12 September 2022) [47], and NCBI Prokaryotic Genome Annotation Pipeline (PGAP) (https://www.ncbi.nlm.nih.gov/genome/annotation_prok/, accessed on 16 February 2021) web services. Functional annotation was carried out using the BlastKoala (https://www.kegg.jp/blastkoala/, v. 2.2, accessed on 15 May 2019) [48] and KEGG (Kyoto Encyclopedia of Genes and Genomes) (https://www.kegg.jp/, accessed on 1 November 2022, release 104.1) [49] databases. Orthology of the genomic regions and prediction of the functional relationships between the genes were determined using the SyntTax service (https://archaea.i2bc.paris-saclay.fr/SyntTax/, accessed on 16 January 2013) [50]. Using the Mauve alignment algorithm (https://doi.org/10.1101/gr.2289704, v. 2.4.0, accessed on 21 December 2014) [51], alignment of the orthologic and xenologic regions was carried out for several genome sequences, which could have been subject to local or large-scale changes (rearrangements, inversions), and the areas free of genome rearrangements (locally collinear blocks–LCBs) were identified. Biosynthetic gene clusters for secondary metabolites were predicted using antiSMASH version 6.1.1 (https://antismash.secondarymetabolites.org/, v. 6.1.1, accessed on 20 January 2022). The phylogenetic tree was constructed by the neighbor-joining method using the iTOL (https://itol.embl.de/, version 6.6, accessed on 22 September 2022) web service with default settings. Genome sequences of strains required for constructing the phylogenetic tree were taken from the NCBI.

### 2.4. Genome Information

The genomes of two bacterial strains, *Gordonia amicalis* 1D and *Gordonia alkanivorans* 135 (VKM Ac-2849D), utilizing DBT as the sole sulfur source, were sequenced and described previously [37,38,39]. The genome of strain 1D is available from GenBank/EMBL/DDBJ under accession number GCA_002327125.1. The genome of strain 135 is available from GenBank/EMBL/DDBJ under accession number GCA_009720185.1, including chromosome (NZ_CP046257.1) and plasmid pG135 (NZ_CP046258.1) sequences.

### 2.5. Nucleotide Sequence Accession Numbers

The GenBank/EMBL/DDBJ accession number of the 16S rRNA gene sequence of strain *Gordonia amicalis* 6-1 is MN101280. The whole-genome project of strain *G. amicalis* 6-1 has been deposited at DDBJ/EMBL/GenBank under the accession number CP096596.1, BioSample number SAMN27756081, BioProject number PRJNA831641.

## 3. Results and Discussion

### 3.1. General Features of the Genomes of Strain G. amicalis 6-1 and Other Dibenzothiophene-Degrading Gordonia Strains

**Figure 1:**
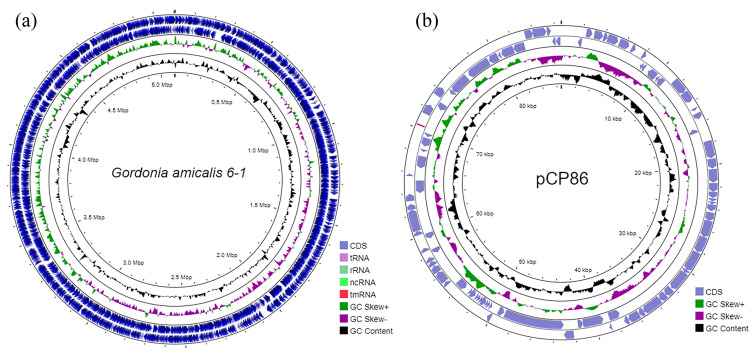
Graphical maps of circular chromosome (**a**) and plasmid pCP86 (**b**) of *G. amicalis* 6-1.

The genome of the strain *G. amicalis* 6-1 was sequenced and completely assembled. This genome consists of a 5,105,798 b.p. circular chromosome with GC content of 67.3% (RefSeq sequence NZ_CP096596.1) and a 86,621 bp circular plasmid pCP86 with GC content of 65.0% (NZ_CP096597.1) (Figure 1, Table 1). Using NCBI PGAP pipeline, in the chromosome 6-1 were predicted 4739 genes, including 4429 protein-coding genes, 9 rRNAs, 47 tRNAs and 3 non-coding RNAs. Table 1 presents the general characteristics of genomes of the strain *G. amicalis* 6-1 and previously described strains *G. amicalis* 1D [37] and *G. alkanivorans* 135 [38,39], utilizing DBT as the sulfur source. Maps of circular chromosomes of strains 1D and 135 and plasmid pG135 are given in Figure S2. To understand the genome structure of DBT-degrading *Gordonia* strains, the order of gene locations in the complete genome sequences of strains 6-1, 1D, and 135 was compared using the Mauve alignment algorithm [51]. Figure S3a shows the homologous regions of the chromosomes.

High similarity between the chromosomes of *G. alkanivorans* 135 and *G. amicalis* 1D, belonging to different species, should be noted. In general, location of the main blocks on the chromosomes of all three strains was similar. However, the central part of the genome of strain 1D contained a single big inversion, while strain 6-1 exhibited small rearrangements, resulting probably from recombination and transposase activity.

For the search of plasmids related to the pCP86 plasmid, the values of average nucleotide identity (ANI) with the plasmids represented in GenBank were determined (Table S1). According to the results of Mauve alignment (Figure S3b) and ANI values, no plasmid which could have belonged to the common ancestor was revealed for pCP86. Comparison of the sequences of CP86 and most closely related plasmids (ANI > 80%) (Figure S3b) revealed rearrangements among the conserved segments, which are represented as colored blocks. For the pG135 plasmid of *G. alkanivorans* strain 135, related plasmids are pYYC01 and pKB1 from *G. alkanivorans* YC-RL2 and *G. westfalica* DSM 44215^T^, respectively.

### 3.2. Taxonomic Assignment of G. amicalis 6-1 Based on Phylogenomic Analysis

**Figure 2:**
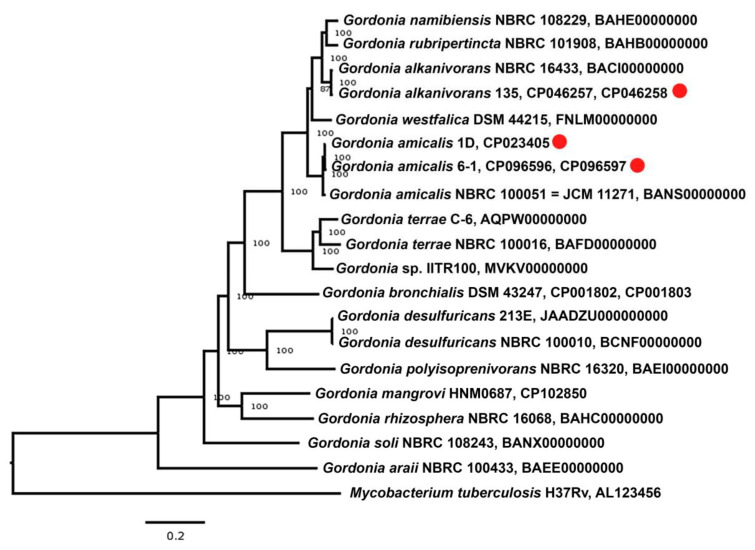
Whole-genome tree of the studied *Gordonia* strains (red markers) and other members of the genus *Gordonia* carried out using the PATRIC [https://www.bv-brc.org/, accessed on 14 September 2022] and iTOL [https://itol.embl.de/, accessed on 22 September 2022] services with default settings. The genome of *Mycobacterium tuberculosis* strain H37Rv (AL123456) was used as an outgroup.

The 16S rRNA gene sequence of strain 6-1 (MH101280) exhibited 99.26% identity and 100% coverage with the gene of the type strain *Gordonia amicalis* IEGM^T^ (JCM 11271 = NBRC 100051), which was previously used to assign strain 6-1 to this species [40]. The 16S rRNA gene sequence of strain 6-1 (MH101280) had also 99.2% identity and 100% coverage with the full gene in the genome of this strain. To specify the species affiliation of strain 6-1, its genome was compared with the genomes of closely related type strains as recommended by Chun et al. [52]. The values for ANI and genomic digital DNA–DNA hybridization (dDDH) for strain 6-1 (Table S2) were 97.7% and 87.9%, respectively, exceeding the values accepted for new species characterization (95–96% and 70%) [52,53,54]. In the phylogenomic tree constructed by the neighbor-joining method using the iTOL service, strains 6-1 and 1D formed a branch with the type strain *G. amicalis* IEGM^T^ within the genus *Gordonia*, which also confirmed their assignment to the species *G. amicalis* (Figure 2).

### 3.3. Functional Characterization of the Genomes of G. amicalis 6-1 and Other Gordonia Strains

**Figure 3:**
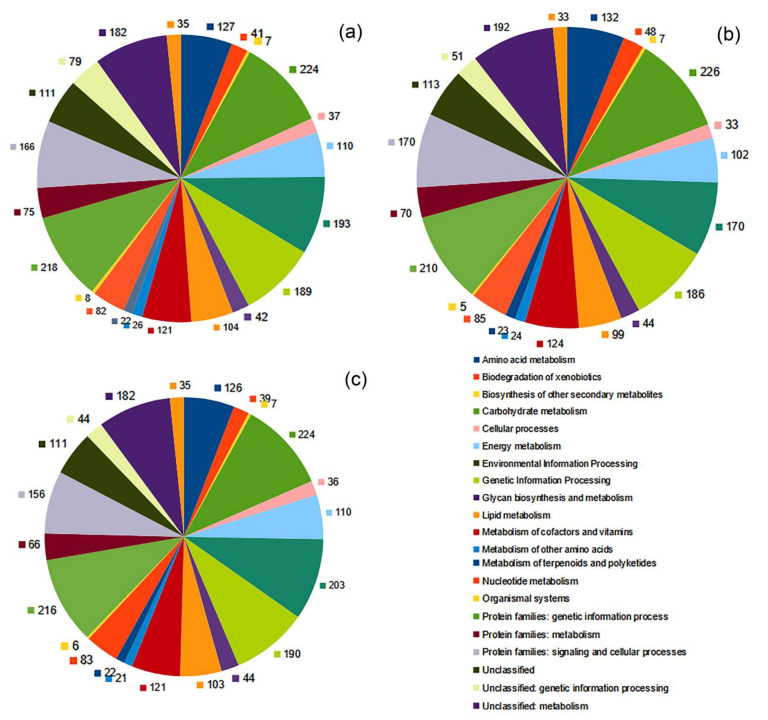
Number of genes associated with general functional categories based on KEGG classification of the chromosomes of strains *G. amicalis* 6-1 (**a**), *G. alkanivorans* 135 (**b**), and *G. amicalis* 1D (**c**).

The coding sequences of the chromosomes and plasmids were annotated and assigned to various functional categories (Figure 3 and Figure S4). In the chromosomes about 47% of the genes were assigned a putative function, while the remaining ones were annotated as hypothetical proteins. Among the predicted products of the genes of the pCP86 plasmid, 49 were analogous to proteins with a known function, 41 were hypothetical; 24 predicted products were assigned to 7 functional pathways. The genes encoding resistance to arsenic (Acr), norfloxacin and enoxacin (MdtH), and adaptation to the media with decreased magnesium content (PhoP) were found. For the pG135 plasmid, 80 products with a known function and 68 with a hypothetical one were predicted. The pG135 plasmid contained eight transposase genes and no *tra* genes, which encode the proteins required for conjugation. The genes for pollutant catabolism and production of secondary metabolites were not revealed in any of the plasmids.

### 3.4. Genetic Systems for Alkane Oxidation

Alkane degradation by members of the genus *Gordonia*, as by members of other genera (for example, *Alcanivorax*, *Dietzia*, *Rhodococcus*, *Nocardia*, *Pseudomonas*) is carried out by alkane hydroxylase enzymes [55,56]. The cluster encoding alkane hydroxylases of the AlkB family usually comprises the *alk*B1 gene encoding alkane-1-monooxygenase (AlkB), the *rub*A1 and *rub*A2 genes encoding rubredoxins, the *rub*R gene encoding rubredoxin reductase and the TetR/AcrR transcription regulator. Bacteria are also known to possess cytochrome P450 monooxygenases involved in *n*-alkane oxidation and belonging to the cytochrome CYP153 family [57]. Similar to AlkB, rubredoxin and rubredoxin reductase are required for the operation of CYP153. It was previously suggested that bacteria contain one of alkane hydroxylases, but it was shown that bacteria of the genera *Alcanivorax*, *Dietzia*, *Rhodococcus*, *Nocardia*, and *Gordonia* possess both AlkB and CYP153 simultaneously, which is possibly an indication of the interaction of these enzyme systems in alkane oxidation [18,55,56,58,59,60,61].

**Figure 4:**
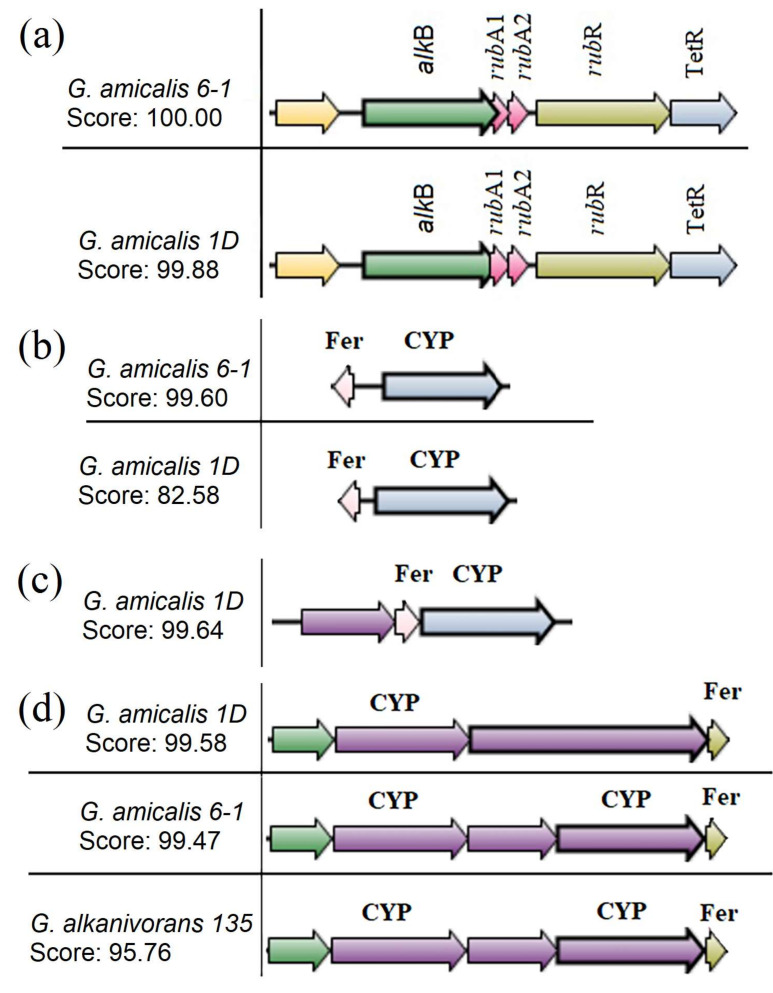
Comparative structure of the genomic clusters encoding alkane hydroxylases AlkB (**a**) and CYP153 (**b**–**d**) families in *Gordonia* strains. Designation: (**b**) strains 6-1 (UPW14530.1) and 1D (ATD70990.1): ferredoxin, cytochrome P450; (**c**) strain 1D (ATD69356.1): oxidoreductase, ferredoxin, cytochrome P450; (**d**) strain 1D (ATD72044.1): TetR family transcriptional regulator, cytochrome P450, hypothetical protein, ferredoxin; strains 6-1 (UPW15792.1) and 135 (QGP89423.1): TetR family transcriptional regulator, cytochrome P450, SDR family oxidoreductase, cytochrome P450, ferredoxin.

The SyntTax service was used to analyze the orthology of the studied genomic regions in strains 6-1, 1D, and 135 (Figure 4). The genomes of *G. amicalis* strains 6-1 and 1D were found to contain all the genes of the alkane hydroxylase cluster of the AlkB family located in one direction (Figure 4a).

In the genome of *G. alkanivorans* 135, the *alk*B gene was not detected. Similar search for the regions encoding monooxygenases of the cytochrome CYP153 family revealed two sites in the genome of strain 6-1 and three sites in the genomes of strains 1d and 135 (Figure 4b–d). A single ferredoxin was usually observed, located collinearly or convergently close to cytochrome P450 (Figure 4b,c). A site was found (Figure 4d), which in strains 6-1 (cytochrome P450 UPW15792.1) and 135 (cytochrome P450 QGP89423.1) comprises co-directed genes encoding TetR family transcriptional regulator, cytochrome P450, SDR family oxidoreductase, cytochrome P450, and ferredoxin. At the same time, strain 1D (cytochrome P450 ATD72044.1) lacks the gene encoding SDR family oxidoreductase, while the gene encoding cytochrome P450 was elongated and was designated as a hypothetical protein, probably due to annotation errors by the service.

According to the KEGG database [49], alkane degradation is included into the “Fatty acid metabolism” pathway (Pathway ID 00071). Monooxygenase enzymes (EC 1.14.15.3) participate in this process both at the initial stage of alkane oxidation and at the terminal stage of ω-oxidation of fatty acids. In the annotated genomes of *G. amicalis* strains 6-1 and 1D, the presence of this enzyme can be inferred for both strains (Figure S5a). In spite of phenomenological observations on growth of *G. alkanivorans* strain 135 on *n*-alkanes, oil, and diesel fuel [39], it contained no monooxygenase enzymes (EC 1.14.15.3) (Figure S5b). These data support the conclusion that alkane-degrading properties of strain 135 result from the presence of CYP153 family alkane hydroxylases.

### 3.5. Genes of Benzoate Metabolism and Degradation of Organosulfur Compound (DBT)

**Figure 5:**
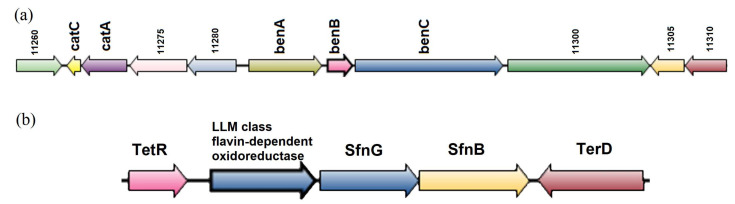
Genomic regions containing benzoate degradation genes (**a**) and the gene *sfn*B (**b**) tentatively involved in DBT desulfurization in *Gordonia* strains.

The genes for the degradation of aromatic compounds were analyzed for all studied strains. Benzoate is a central metabolite in the degradation of various aromatic compounds. In the benzoate degradation subcategory, the genes were revealed which belonged to 18 orthologic groups, including the catechol metabolism genes *cat*A (catechol 1,2-dioxygenase) and *cat*C (muconolactone D-isomerase). Analysis of the region showed that the *cat*AC genes were located convergently relative to *ben*ABC (Figure 5a). The genes responsible for the degradation of other most common polyaromatic hydrocarbons (the *nar* for naphthalene and the *nid* и *phd* operons for phenanthrene) were not detected (Table S3).

Figure S6 shows the “Benzoate degradation via the hydroxylation” pathway (ID 00362) and indicates the enzymes involved. The genome of *G. amicalis* 6-1 contains the genes that encode enzymes of aerobic benzoate transformation into catechol: benzoate 1,2-dioxygenase (EC 1.14.12.10) and dihydroxycyclohexadiene dehydrogenase (EC 1.3.1.25). Its genome was found to possess the genes encoding enzymes of *ortho*-degradation of catechol to 3-oxoadipate. This pathway of benzoate degradation was similar to those in strains 1D and 135.

Search for genetic structures responsible for biodesulfurization revealed that sulfur metabolism is controlled by 22 genes in *G. alkanivorans* 135 and by 24 genes in *G. amicalis* 1D and 6-1. Although the strains were capable of using DBT as the sole sulfur source, no genes of the dsz operon were revealed by the BLAST service (Table S4).

Search for the genes related to *dsz*, which could probably determine the process of DBT catabolism was carried out. Some services reported the presence of the *dszC* in the annotated genomes of the studied strains (Table 2).

According to the InterPro database (https://www.ebi.ac.uk/interpro, accessed on 15 December 2022) [62], the acyl-CoA dehydrogenase gene is close to the SfnB family genes responsible for sulfur assimilation, while *sfn*B is related to the *dsz*C gene responsible for DBT desulfurization. All detected genes were examined using SyntTax. Analysis of the SfnB-containing sequence revealed a conserved region in the strains, for which normalization compared to the standard exceeded 70% (Figure 5b).

The species *G. alkanivorans*, *G. amicalis*, *G. rubripertincta*, *G. jinghuaiqii*, *G. hongkongensis*, and *G. terrae* are characterized by the following structure of the studied region: a regulator of the TetR family, LLM class flavin-dependent oxidoreductase, dimethyl sulfone monooxygenase SfnG, SfnB family sulfur acquisition oxidoreductase, and convergently positioned TerD (also designated as a stress protein). The TetR regulator family is one of the largest and best-studied groups of the prokaryotic single-component signal transduction system. They are involved in the regulation of the genes responsible for various catabolic and anabolic processes, including the regulation of osmotic stress [63]. According to the literature data, a protein of the TetR family designated DszGR, is involved in regulation of the dsz operon in *Gordonia* sp. strain IITR100 [64]. The mechanism of regulation by this protein was also investigated [65]. The authors reported specific binding of the DszGR protein to upstream sequences, inducing a bend required for activity of the dsz promoter. The UniProt database (https://www.uniprot.org/, accessed on 31 October 2022) [66] contains the information on relations between LLM class flavin-dependent oxidoreductase and DszA and mentions such alternative names as alkane sulfonate monooxygenase, SsuD, and FMNH_2_-dependent monooxygenase. Although the similarity of this enzyme to the translated sequence of the *dsz*A gene product is relatively low, it performs the same functions, i.e., it catalyzes the reactions requiring FMNH_2_ and O_2_ with FMN release. SfnG is known to convert dimethyl sulfone to methane sulfinate [67]. This process occurs in the second reaction of the 4S pathway, where DBT sulfone is converted to 2-hydroxybiphenyl-2-sulfinate. Thus, it may be concluded that SfnG is probably involved in the reaction similar to the 4S involving DszC.

Analysis of our data supports the suggestion that the region encoding TetR, LLM class flavin-dependent oxidoreductase, SfnG, SfnB, and TerD may determine DBT degradation by bacteria lacking the *dsz* genes in their genomes.

### 3.6. Genes of Osmoprotectant Metabolism

**Figure 6:**
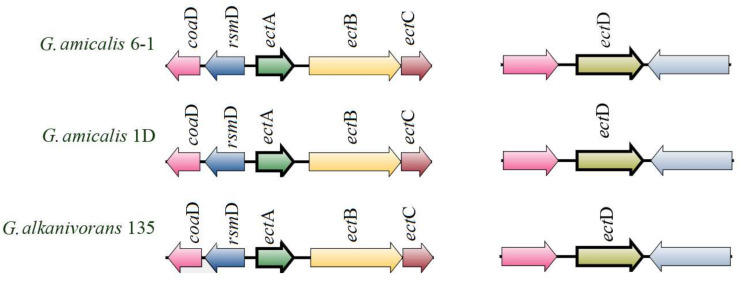
Genomic regions containing ectoine biosynthesis genes in *Gordonia* strains 6-1, 1D, and 135.

The strains *G. amicalis* 6-1 and 1D and *G. alkanivorans* 135 grew at high salinity with the NaCl optima at 1.5–7.0%, 1.0–3.0%, and 1% (*w*/*v*) NaCl, respectively. Search for the genes responsible for osmoprotectant (betaine and ectoine) production in the genomes of the studied *Gordonia* was carried out. In the KEGG database, osmoprotectors synthesis is included in the “Glycine, serine and threonine metabolism” pathway (ID 00260; Figure S7). A complete pathway for biosynthesis of ectoine and 5-hydroxyectoine was revealed in the genomes of all three strains. The cluster for production of ectoine, an osmolyte produced and released in response to salt stress variations, contains the *ect*A gene encoding L-2,4-diaminobutyric acid acetyltransferase, the *ect*B gene encoding diaminobutyrate-2-oxoglutarate transaminase, and *ect*C encoding L-ectoine synthase (Figure 6). The separately located *ect*D (ectoine hydroxylase) gene was also found in the genomes of the studied strains. Thus, in *G. amicalis* strain 6-1 it is located between the genes encoding PaaI family thioesterase and CoA transferase, and in strains *G. amicalis* 1D and *G. alkanivorans* 135, between the genes encoding PaaI family thioesterase and 2-methylfumaryl-CoA isomerase.

Genome analysis using the antiSMASH service revealed a region determining ectoine biosynthesis in the genomes of strains 6-1, 1D, and 135. The ectoine cluster of strains 6-1 and 1D contains 75% genes showing similarity with the ectoine cluster genes in the database. The highest similarity (up to 100%) was usually observed within a single genus (Figure S8).

Among the genes of betaine formation, each strain was found to contain two copies of the *bet*B (betaine-aldehyde dehydrogenase) gene and one copy of *bet*T (betaine transport protein). The *bet*T gene controls osmotic adaptation and betaine transport from the environment; sorption requires less energy expenditure than biosynthesis [68]. While the *bet*A gene was not revealed, the *cod*A was found, which encoded choline oxidase [EC 1.1.3.17], an enzyme with a similar function to *bet*A (Figure S7).

Genome analysis and the results of phenotypic research on *G. amicalis* strain 6-1 indicate its adaptation to the conditions of the habitat and ability to degrade oil *n*-alkanes at high salinity and to use organosulfur compounds as sulfur sources. The high metabolic potential of *G. amicalis* strain 6-1 and of dibenzothiophene-desulfurizing strains *G. amicalis* 1D and *G. alkanivorans* 135 makes it possible to apply them in environmental biotechnologies aimed at bioremediation of oil-contaminated environments and for removal of oil and asphalt-resin-paraffin deposits from the well bottom zone and for microbial enhancement of oil recovery.

## 4. Conclusions

The complete genome sequence of *Gordonia amicalis* 6-1 provides valuable insights into the metabolic potential of a novel hydrocarbon-oxidizing, dibenzothiophene-desulfurizing strain of the genus *Gordonia*. Genome analysis of *G. amicalis* 6-1 and KEGG reconstruction results indicated a range of functional genes involved in strain adaptation to the environment–utilization of alkanes and aromatic compounds as carbon and energy sources and of dibenzothiophene as a sulfur source, as well as biosynthesis of osmolites betaine and ectoine, enabling growth at high ambient salinity. Dibenzothiophene-desulfurizing *Gordonia* strains 6-1, 1D, and 135 cannot use the 4S pathway encoded by the dszABC operon-like structure for assimilation sulfur from condensed thiophenes. It is suggested that the genome region encoding TetR, LLM class flavin-dependent oxidoreductase, SfnG, SfnB, and TerD, is responsible for dibenzothiophene degradation in bacteria lacking the *dsz* genes in their genomes. Genome analysis of the strains and phenomenological studies exhibited considerable potential of their applications in bioremediation of oil-contaminated environments and degradation of asphalt-resin-paraffin deposits in petroleum industry.

## 5. Patents

Borzenkov, I.A.; Sokolova, D.Sh.; Nazina, T.N.; Babich, T.L.; Semenova, E.M.; Ershov, A.P.; Khisametdinov, M.R. *Gordonia amicalis* strain with ability of generation directly in oil reservoir of oil-displacing agent—biopav and decreasing content of organosulfur compounds of oil. Registration date: 29.11.2018. Patent RU 2 673,747 C1. http://www1.fips.ru/fips_servl/fips_servlet?DB=RUPAT&DocNumber=2673747&TypeFile=html.

## Figures and Tables

**Table 1:** Characteristics of the genome sequences (chromosome and plasmid) of hydrocarbon-oxidizing dibenzothiophene-degrading bacteria of the genus *Gordonia* used in this study.

Property	*G. amicalis* 6-1	*G. amicalis* 1D	*G. alkanivorans* 135
Chromosome			
Assembly Accession no.	GCA_023238565.1	GCA_002327125.1	GCA_009720185.1
Chromosome size (bp)	5,105,798	5,151,623	5,039,827
G + C content (%)	67.4	67.3	67.4
Genes (Total)	4739	4677	4704
Protein-coding genes	4429	4448	4492
Total RNAs	59	59	66
tRNAs	47	47	51
ncRNAs	3	3	3
Pseudo Genes	251	170	146
Closest Placement	*G. amicalis* NBRC 100051	*G. amicalis* NBRC 100051	*G. alkanivorans* NBRC 16433
ANI/dDDH	97.7%/87.9%	98.1%/88.6%	98.4%/86.8%
Plasmid	pCP86		pG135
Accession no.	CP096597.1		CP046258.1
Total length	86,621		164,963
G + C content (%)	65.0		64.5

**Table 2:** Gene products annotated as DszC.

Strain	Encoded Product	GeneBank no.
*G. amicalis* 6-1	SfnB	UPW13782.1
	oxidoreductase	UPW14477.1
	acyl-CoA dehydrogenase	UPW14265.1
	acyl-CoA dehydrogenase	UPW15449.1
*G. alkanivorans* 135	SfnB	QGP87489.1
	acyl-CoA dehydrogenase	QGP87979.1
	acyl-CoA dehydrogenase	QGP89059.1
*G. amicalis* 1D	SfnB	ATD70150.1
	oxidoreductase	ATD71050.1
	acyl-CoA dehydrogenase	ATD71233.1
	acyl-CoA dehydrogenase	ATD71690.1

## Data Availability

The GenBank/EMBL/DDBJ accession number of the 16S rRNA gene sequence of strain *Gordonia amicalis* 6-1 is MN101280. The whole-genome project of strain *G. amicalis* 6-1 has been deposited at DDBJ/EMBL/GenBank under the accession number CP096596.1, BioSample number SAMN27756081, BioProject number PRJNA831641.

## Supplementary Materials

The following supporting information can be downloaded at: https://www.mdpi.com/article/10.3390/microorganisms11010004/s1, Figure S1: Growth of *G. amicalis* strain 6-1 in an oil-containing medium (left) compared to the uninoculated control (right) (a); localization of cells inside oil droplets in a liquid medium (an Axio Imager.D1 microscope, Carl Zeiss, Germany) (b); micrograph of negatively stained cells of strain 6-1 in a JEM-100CX transmission electron microscope (JEOL Ltd., Tokyo, Japan) (c). The cells were grown in medium with crude oil at 28 °C for 7 days. Scale bar corresponds to 1 µm. Figure S2: Graphical maps of circular chromosomes of strains *G. amicalis* 1D (a) and *G. alkanivorans* 135 (b) and of circular plasmide pG135 of strain *G. alkanivorans* 135 (c). Figure S3: Whole genome comparative Mauve alignment of chromosomes of *G. alkanivorans* 135, *G. amicalis* 1D, *G. amicalis* 6-1 (a); and plasmids pYYC01 (*G. alkanivorans* YC-RL2), pCP86 (*G. amicalis* 6-1), p174 (*G. polyisoprenivorans* VH2), and pPE105 (*G. terrae* PE42) (b). Colored blocks indicate genome regions aligned with parts of another genome. Each sequence of identically colored blocks represents a collinear set of matching. Homologous blocks in different genomes are connected with lines. Conservation level of genomic regions is shown by the height of the similarity profile. Figure S4: Number of genes associated with general functional categories based on KEGG classification of the plasmids pCP86 (*G. amicalis* 6-1) (a) and pG135 (*G. alkanivorans* 135) (b). Figure S5: Predicted enzyme profile of the fatty acid metabolism pathway (ID 00071) in *G. amicalis* 6-1 (a) and *G. alkanivorans* 135 (b) according to the KEGG database. Figure S6: Predicted enzyme profile of the benzoate degradation via the hydroxylation pathway (ID 00362) in *G. amicalis 6-1* according to the KEGG database. Figure S7: Predicted enzyme profile of betaine and ectoine biosynthesis pathways in *G. amicalis* 6-1 according to the KEGG database and the genes encoding the biosynthesis of these osmoprotectors in the genomes of strains 6-1, 1D and 135. Figure S8: The ectoine biosynthesis gene cluster in genomes of *Gordonia* spp. and relative genera based on the antiSMASH analysis. The percentage of genes that show similarity to the genes of the ectoine cluster of *G. amicalis* 6-1, *G. amicalis* 1D, and *G. alkanivorans* 135 is indicated in parentheses. Table S1: Average nucleotide identity (ANI) values (%) for plasmid pCP86 from *G. amicalis* 6-1 and phylogenetically related plasmids from GenBank. Table S2: Average nucleotide identity (ANI) and digital DNA–DNA hybridization (dDDH) values (%) for *Gordonia* strains 6-1, 1D, and 135 and the type strains of the phylogenetically related species of the genus *Gordonia*. Table S3: Genes for degradation of aromatic compounds found in the genomes of *G. amicalis* 6-1, *G. amicalis* 1D, and *G. alkanivorans* 135 based on the annotation from PATRIC online server. Table S4: Genes of the dsz operon found in genomes of *Gordonia* strains using Blast*.
