# Supplementary material for: Comparative Genomic Analysis of the Hydrocarbon-Oxidizing Dibenzothiophene-Desulfurizing Gordonia Strains
Source: Microorganisms. 2022 Dec 20;11(1):4. doi: 10.3390/microorganisms11010004 (PMC9861168; doi:10.3390/microorganisms11010004)
Supplement: Supplementary file 1 [file microorganisms-11-00004-s001.zip › microorganisms-2095014-supplementary.pdf]

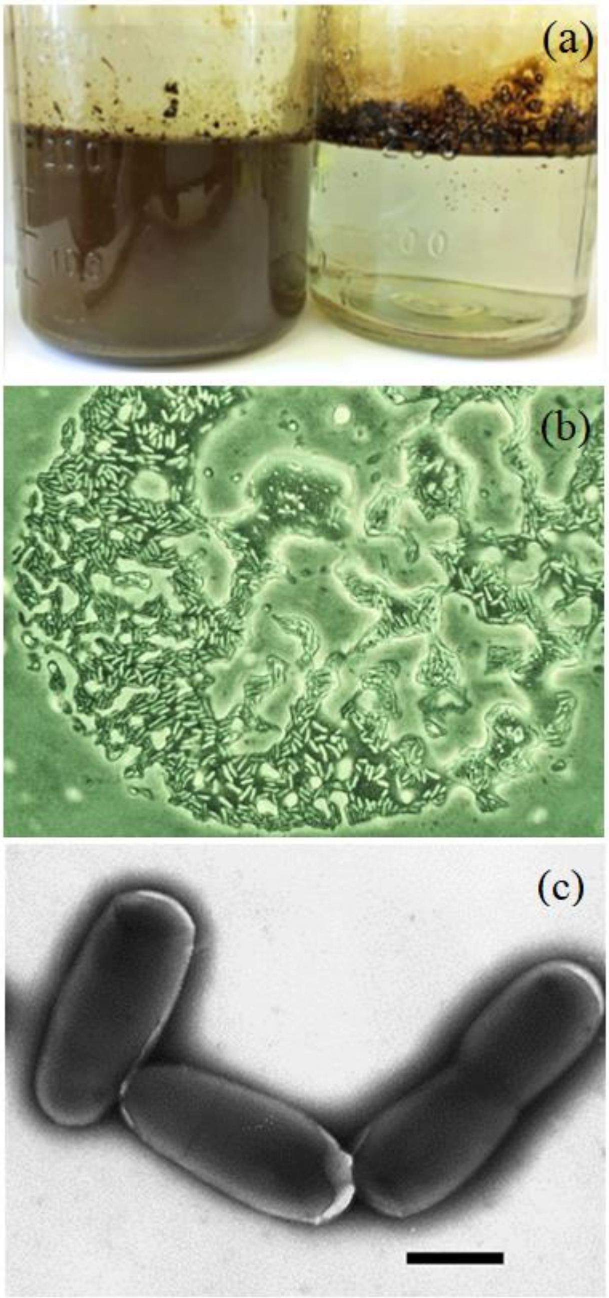

Figure S1. Growth of *G. amicalis* strain 6-1 in an oil-containing medium (left) compared to the uninoculated control (right) (a); localization of cells inside oil droplets in a liquid medium (an Axio Imager.D1 microscope, Carl Zeiss, Germany) (b); micrograph of negatively stained cells of strain 6-1 in a JEM-100CX transmission electron microscope (JEOL Ltd., Tokyo, Japan) (c). The cells were grown in medium with crude oil at 28 °C for 7 days. Scale bar corresponds to 1 μm.

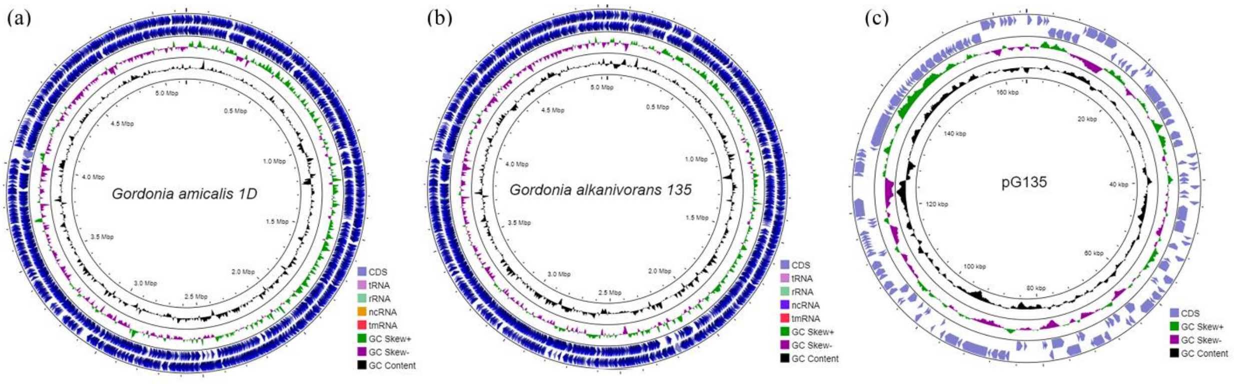

Figure S2. Graphical maps of circular chromosomes of strains *G. amicalis* 1D (a) and *G. alkanivorans* 135 (b) and of circular plasmide pG135 of strain *G. alkanivorans* 135 (c).

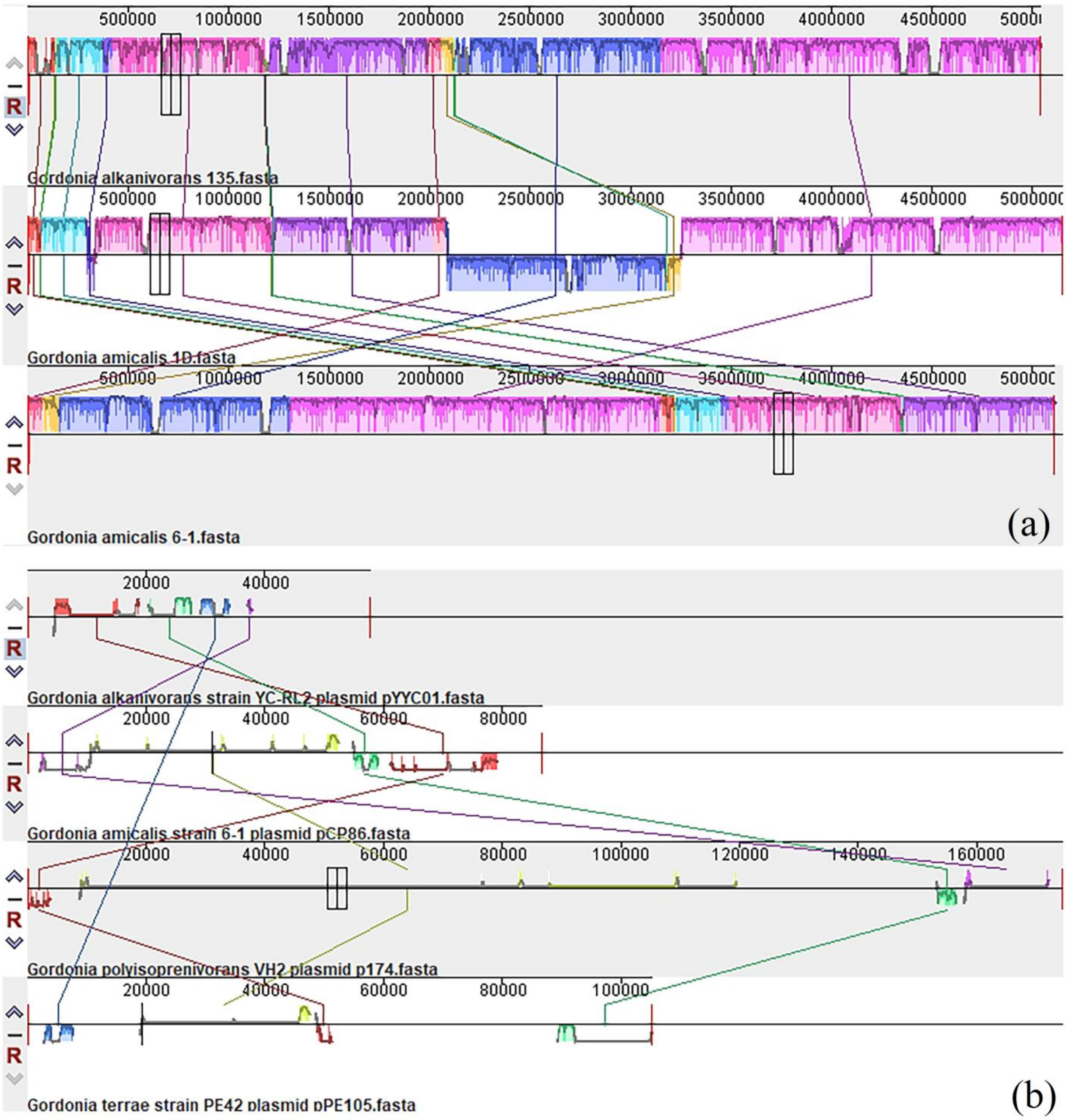

Figure S3. Whole genome comparative Mauve alignment of chromosomes of *G. alkanivorans* 135, *G. amicalis* 1D, *G. amicalis* 6-1 (a); and plasmids pYYC01 (*G. alkanivorans* YC-RL2), pCP86 (*G. amicalis* 6-1), p174 (*G. polyisoprenivorans* VH2), and pPE105 (*G. terrae* PE42) (b). Colored blocks indicate genome regions aligned with parts of another genome. Each sequence of identically colored blocks represents a collinear set of matching. Homologous blocks in different genomes are connected with lines. Conservation level of genomic regions is shown by the height of the similarity profile.

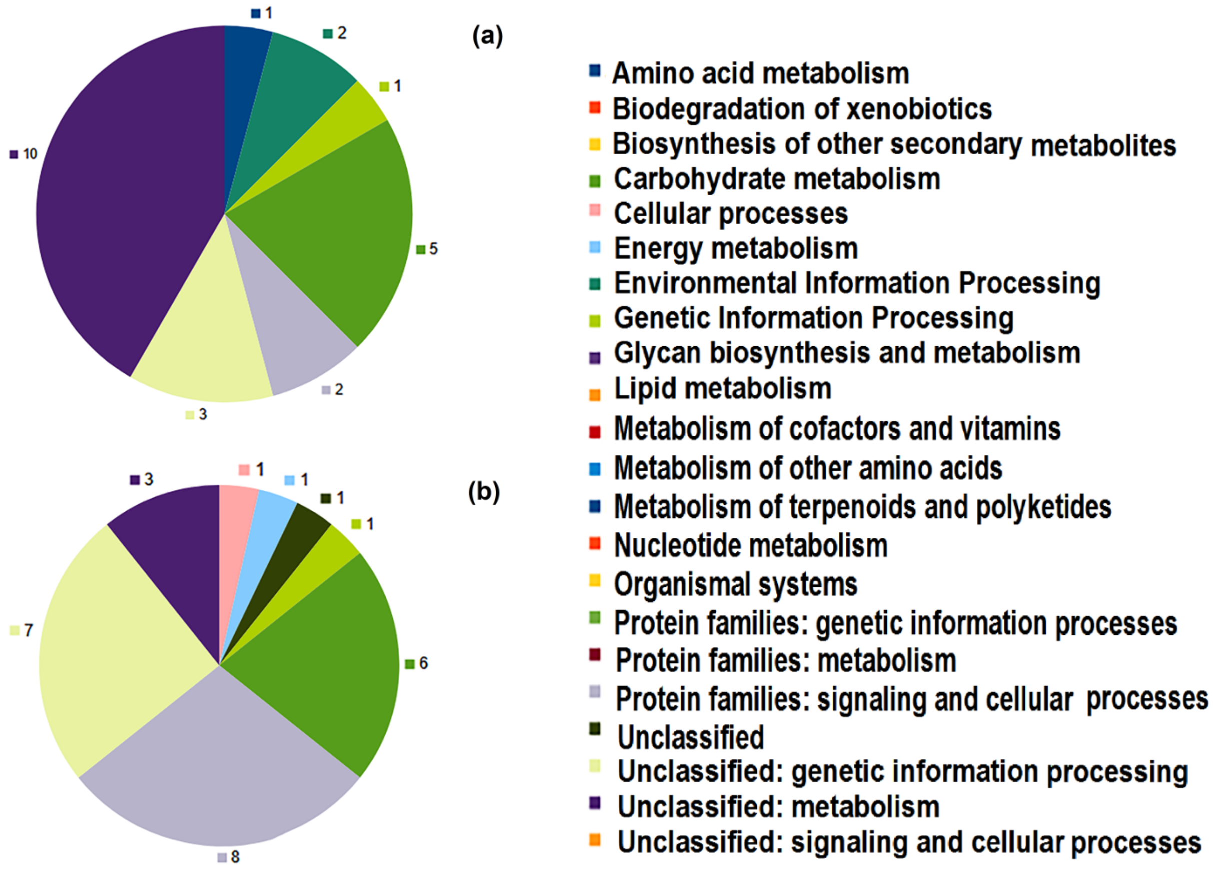

Figure S4. Number of genes associated with general functional categories based on KEGG classification of the plasmids pCP86 (*G. amicalis* 6-1) (a) and pG135 (*G. alkanivorans* 135) (b).

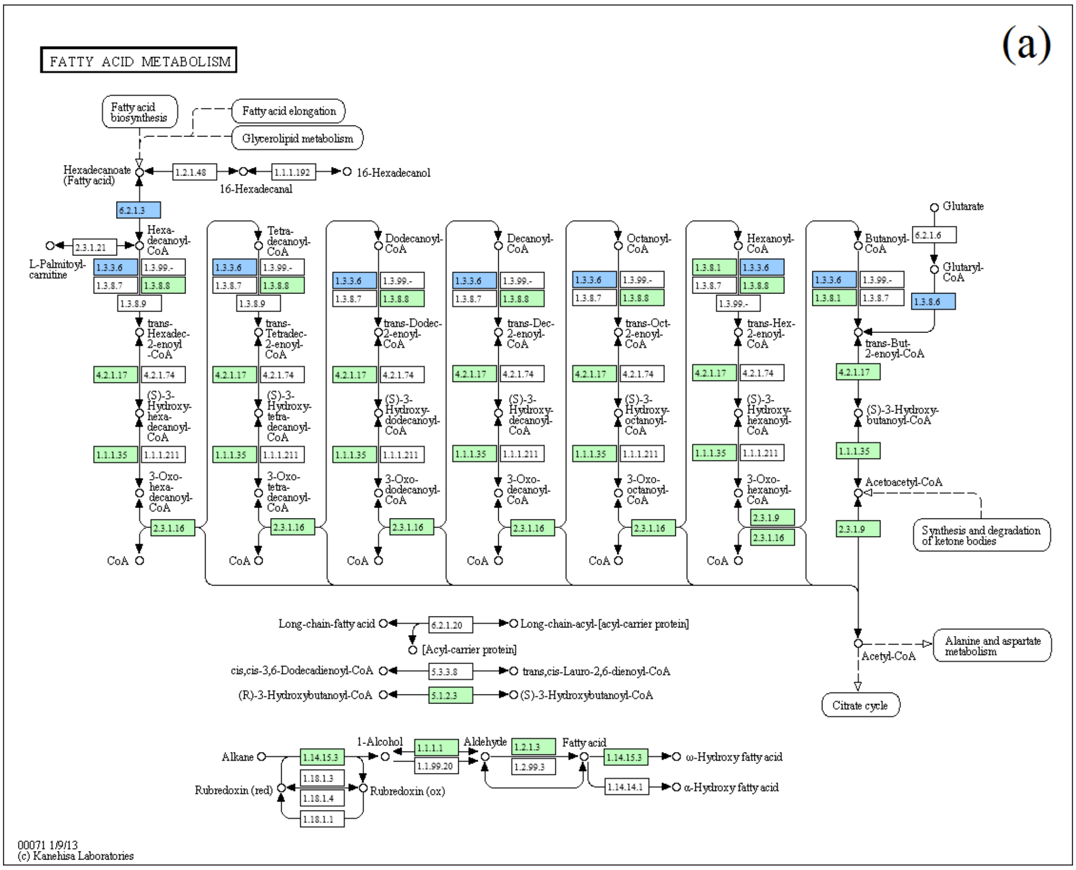

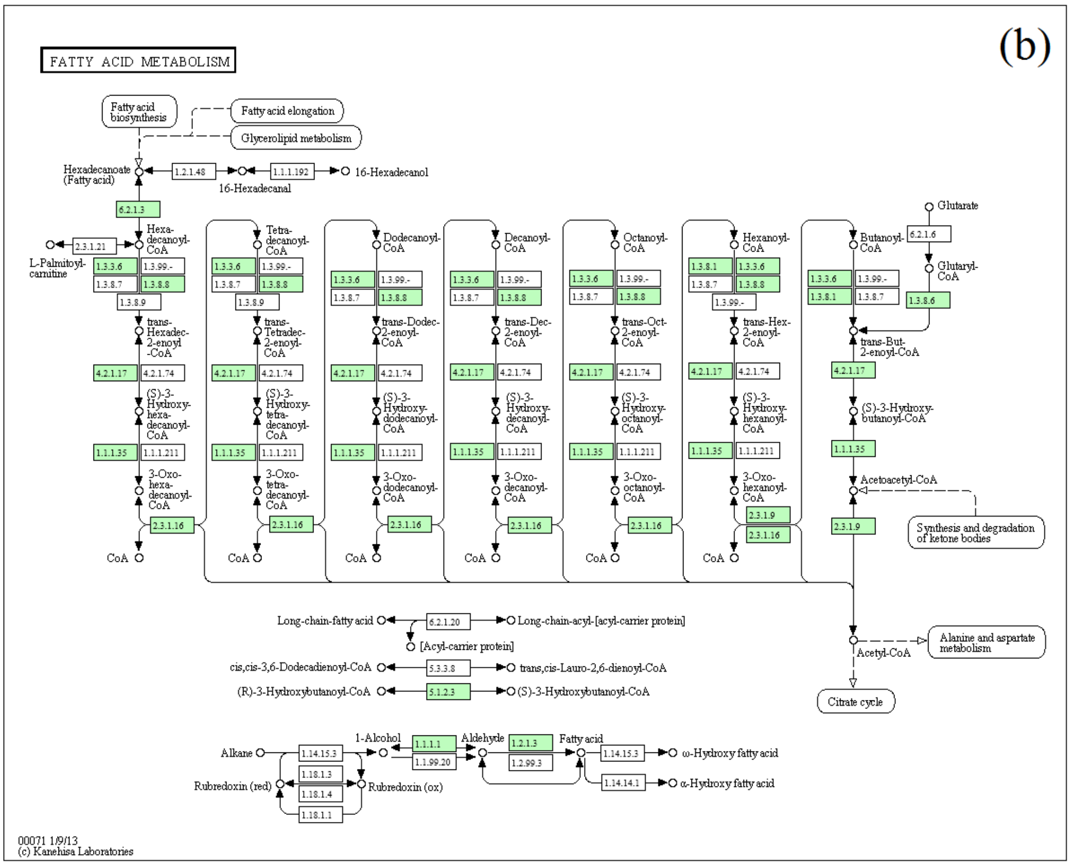

Figure S5. Predicted enzyme profile of the “Fatty acid metabolism” pathway (ID 00071) in *G. amicalis* 6-1 (a) and *G. alkanivorans* 135 (b) according to the KEGG database.

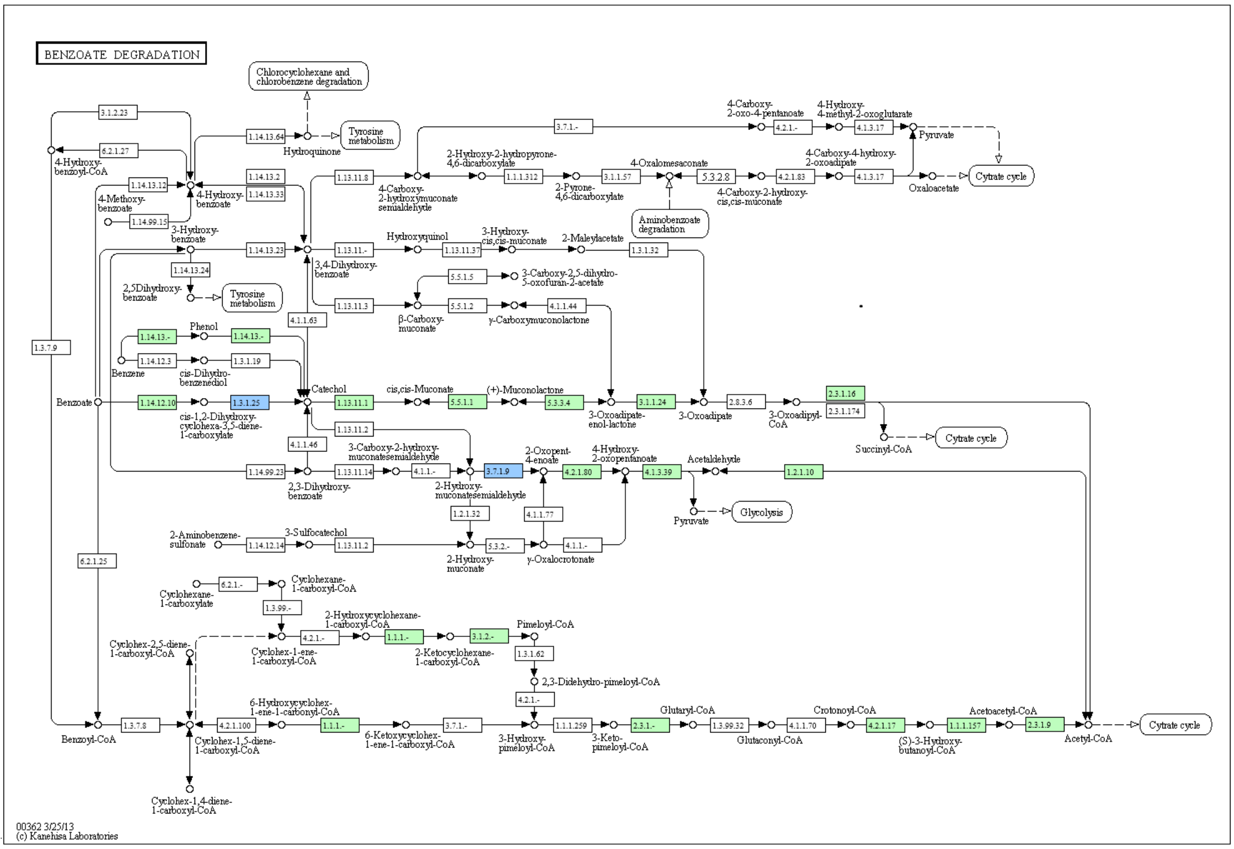

Figure S6. Predicted enzyme profile of the "Benzoate degradation via the hydroxylation" pathway (ID 00362) in *G. amicalis* 6-1 according to the KEGG database.

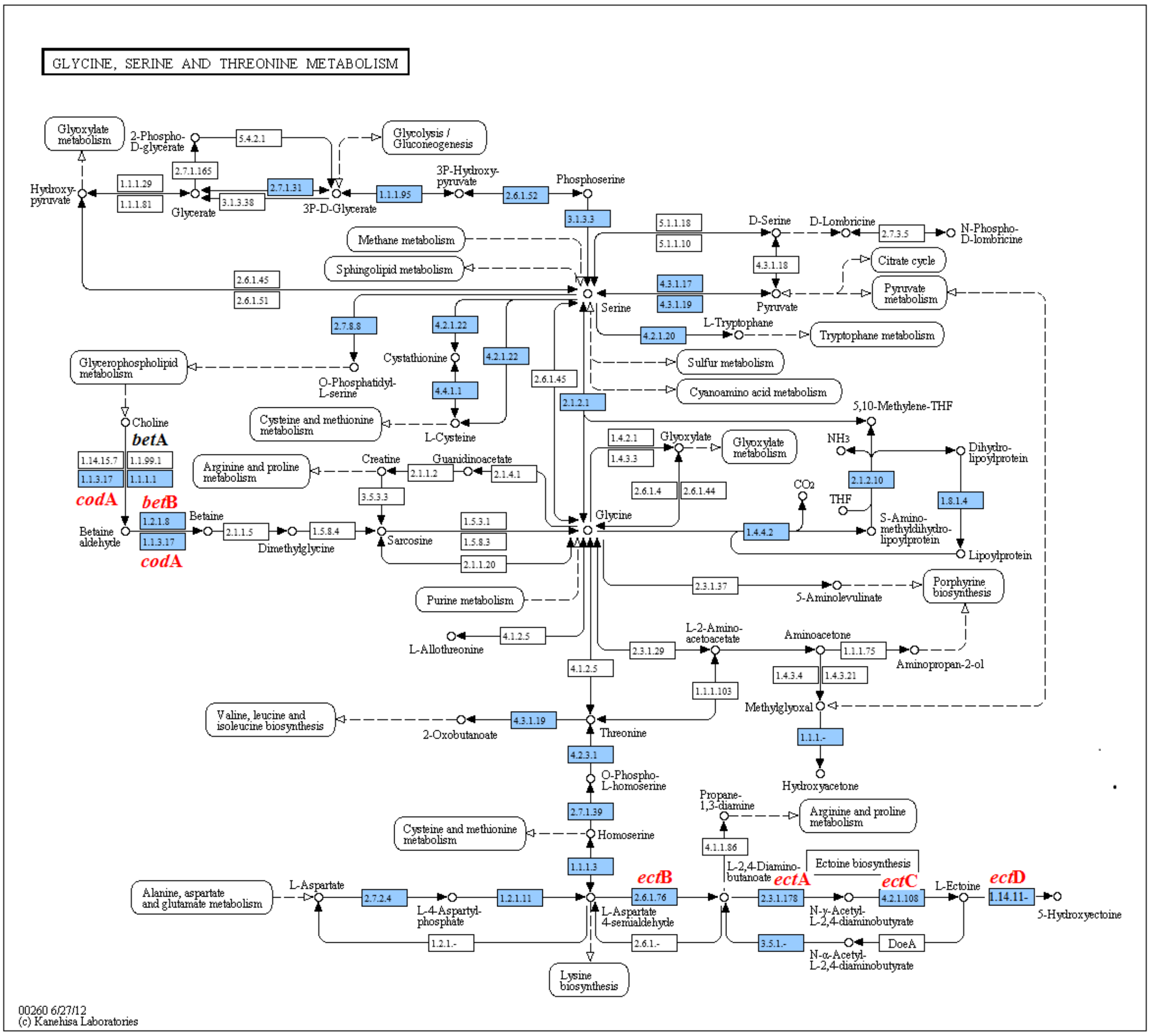

Figure S7. Predicted enzyme profile of "Glycine, serine and threonine metabolism" pathway (ID 00260) and of betaine and ectoine biosynthesis pathways in *G. amicalis* 6-1 according to the KEGG database and the genes encoding the biosynthesis of these osmoprotectants in the genomes of strains 6-1, 1D and 135.

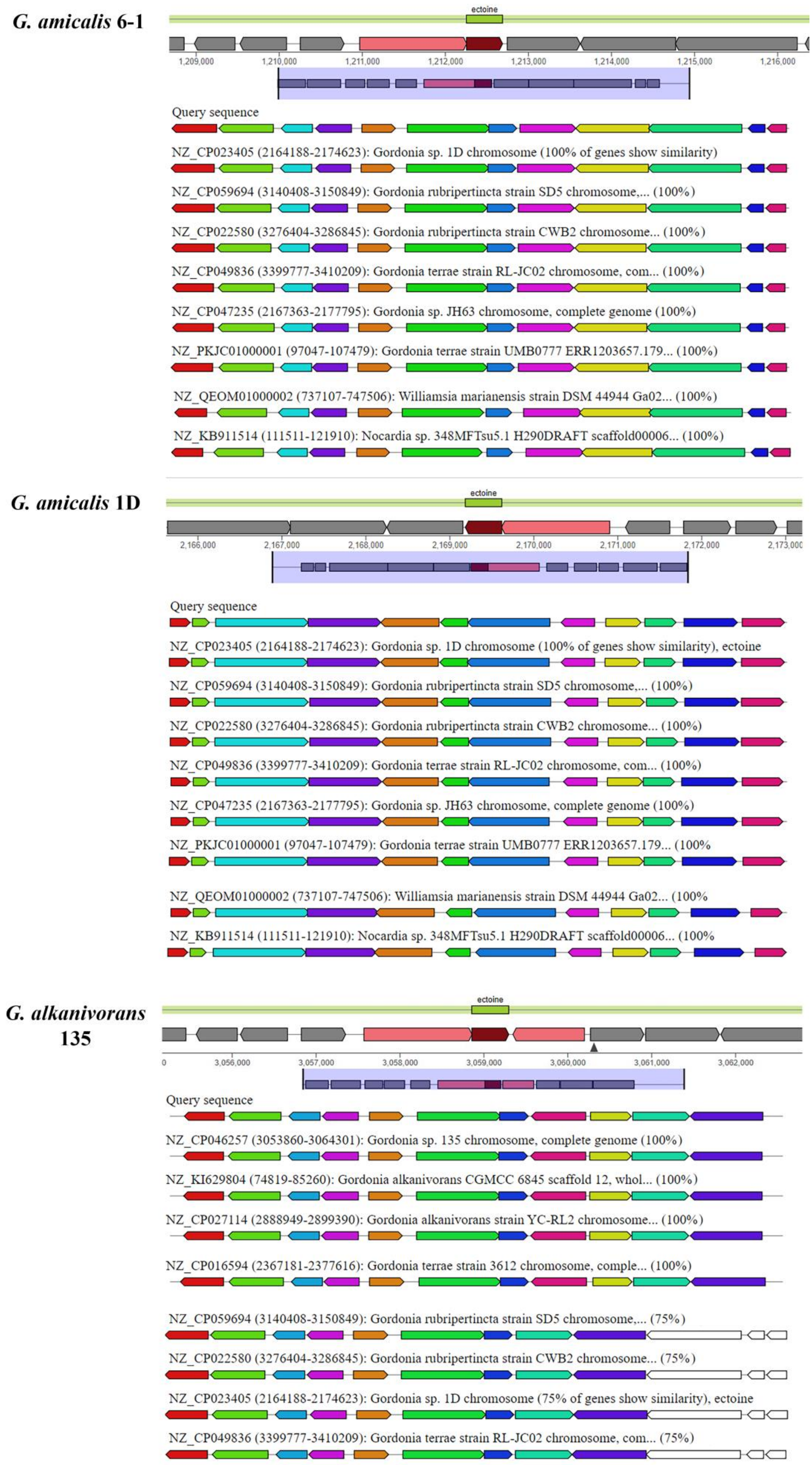

Figure S8. The ectoine biosynthesis gene cluster in genomes of *Gordonia* spp. and relative genera based on the antiSMASH analysis. The percentage of genes that show similarity to the genes of the ectoine cluster of *G. amicalis* 6-1, *G. amicalis* 1D, and *G. alkanivorans* 135 is indicated in parentheses.

Table S1. Average nucleotide identity (ANI) values (%) for plasmid pCP86 from *G. amicalis* 6-1 and phylogenetically related plasmids from GenBank.

Strain	ANI, %	GenBank Number
Gordonia alkanivorans strain YC-RL2 plasmid pYYC01	87.05	NZ_CP027115.1
Gordonia terrae strain PE42 plasmid pPE105	85.90	NZ_CP096586.1
Gordonia polyisoprenivorans VH2 plasmid p174	81.70	NC_016907.1
Gordonia rubripertincta strain CWB2 plasmid pGCWB2	79.77	NZ_CP022581.1
Gordonia sp. KTR9 plasmid pGKT1	77.29	NC_018582.1
Gordonia westfalica strain DSM44215T plasmid pKB1	75.72	NC_005307.1
Gordonia alkanivorans 135 plasmid pG135	74.69	NZ_CP046258.1
Gordonia rubripertincta strain SD5 plasmid pGRS1	73.22	NZ_CP059695.1
Gordonia pseudoamarae strain BEN371 plasmid p1	68.91	NZ_CP045807.1
Gordonia sp. KTR9 plasmid pGKT3	66.66	NC_018583.1
Gordonia bronchialis DSM 43247 plasmid pGBRO01	64.00	NC_013442.1
Gordonia hongkongensis strain JCM 31934 plasmid pGo-1	62.60	NZ_CP095553.1

Table S2. Average nucleotide identity (ANI) and digital DNA–DNA hybridization (dDDH) values (%) for *Gordonia* strains 6-1, 1D, and 135 and the type strains of the phylogenetically related species of the genus *Gordonia.*

Type strain	ANI			dDDH		
	**6-1**	**1D**	**135**	**6-1**	**1D**	**135**
Gordonia amicalis NBRC 100051	97.7	98.1	88.2	87.9	88.6	68.1
Gordonia alkanivorans NBRC 16433	88.4	88.2	98.4	68.1	68.7	86.8
Gordonia rubripertincta NBRC 101908	88.4	88.1	92.5	66.9	64.4	72.6
Gordonia bronchialis DSM 43247	77.4	77.4	77.5	22.1	22.3	22.5
Gordonia terrae NBRC 100016	81.0	80.9	80.6	37.1	38.1	35.6

Table S3. Genes for degradation of aromatic compounds found in the genomes of *G. amicalis* 6-1, *G. amicalis* 1D, and *G. alkanivorans* 135 based on the annotation from Patric database.

Pathway	Gene	Strain		
		G. amicalis **1D**	***G. amicalis*** **6-1**	***G. alkanivorans*** **135**
Benzoate degradation	*benA*	+	+	+
	benB	+	+	+
	benC	+	+	+
	benD	-	-	-
	catA	+	+	+
	catB	-	-	-
	catC	+	+	+
	catD	-	-	-
Naphthalene degradation	*narR1*	-	-	-
	narR2	-	-	-
	narAa	-	-	-
	narAb	-	-	-
	narB	-	-	-
	narC	-	-	-
Phenanthrene degradation	*nidA*	-	-	-
	nidB	-	-	-
	nidD	-	-	-
	phdE	-	-	-
	phdF	-	-	-
	phdG	-	-	-
	phdI	-	-	-
	phdJ	-	-	-

Table S4. Genes of the dsz operon found in genomes of *Gordonia* strains using Blast*.

Strain	Genes					
	***dsz*A**		***dsz*B**		***dsz*C**	
	**Query Cover**	**Percent Identity**	**Query Cover**	**Percent Identity**	**Query Cover**	**Percent Identity**
G. amicalis 6-1	57	71.43	13	95.65	19	77.14
G. amicalis 1D	45	71.43	6	95.65	17	77.14
G. alkanivorans 135	25	74.26	4	89.29	12	75.71

*Query Cover и Percent Identity, parameters for comparing the requested sequences in BLAST.
